# Incidence and cost of vertebral fracture in urban China: a 5-year population-based cohort study

**DOI:** 10.1097/JS9.0000000000000411

**Published:** 2023-05-03

**Authors:** Xuan-Qi Zheng, Lu Xu, Jie Huang, Cheng-Gui Zhang, Wan-Qiong Yuan, Chui-Guo Sun, Zhi-Shan Zhang, Chen Wei, Jin-Xi Wang, Steven R. Cummings, Wei-Bo Xia, Sheng-Feng Wang, Si-Yan Zhan, Chun-Li Song

**Affiliations:** a Department of Orthopaedics; b Research Center of Clinical Epidemiology, Peking University Third Hospital; c Key Laboratory of Epidemiology of Major Diseases (Peking University), Ministry of Education, Beijing; d Beijing Key Laboratory of Spinal Disease Research, Engineering Research Center of Bone and Joint Precision Medicine, Beijing; e Shanghai Songsheng Business Consulting Co. LTD, Shanghai, China; f San Francisco Coordinating Center, California Pacific Medical Center Research Institute, San Francisco, California, USA; g Department of Endocrinology, Key Laboratory of Endocrinology, Ministry of Health, Peking Union Medical College Hospital, Peking Union Medical College and Chinese Academy of Medical Science; h Department of Epidemiology and Biostatistics, School of Public Health, Peking University; i Center for Intelligent Public Health, Institute for Artificial Intelligence, Peking University, Beijing, China

**Keywords:** cost of illness, epidemiology, incidence, osteoporosis, vertebral fracture

## Abstract

Osteoporotic vertebral fractures cause pain and disability, which result in a heavy socioeconomic burden. However, the incidence and cost of vertebral fractures in China are unknown. We aimed to assess the incidence and cost of clinically recognized vertebral fractures among people aged 50 years and older in China from 2013 to 2017.

**Materials and methods:**

This population-based cohort study was conducted by using Urban Employee Basic Medical Insurance (UEBMI) and Urban Resident Basic Medical Insurance (URBMI) data in China from 2013 to 2017, which covered more than 95% of the Chinese population in urban areas. Vertebral fractures were identified by the primary diagnosis (i.e. International Classification of Diseases code or text of diagnosis) in UEBMI and URBMI. The incidence and medical cost of these clinically recognized vertebral fractures in urban China were calculated.

**Results:**

A total of 271 981 vertebral fractures (186 428, 68.5% females and 85 553, 31.5% males) were identified, with a mean age of 70.26 years. The incidence of vertebral fractures among patients aged 50 years and over in China increased ~1.79-fold during the 5 years, from 85.21 per 100 000 person-years in 2013 to 152.13 per 100 000 person-years in 2017. Medical costs for vertebral fractures increased from US$92.74 million in 2013 to US$505.3 million in 2017. Annual costs per vertebral fracture case increased from US$3.54 thousand in 2013 to US$5.35 thousand in 2017.

**Conclusion:**

The dramatic increase in the incidence and cost of clinically recognized vertebral fractures among patients aged 50 and over in urban China implies that more attention should be given to the management of osteoporosis to prevent osteoporotic fractures.

## Introduction

Highlights

- The incidence and medical cost of vertebral fracture in China is increasing.
- The incidence of vertebral fracture in China peaked at 80+ years of age.
- The incidence in females was nearly three times higher than that in males.
- The reasons for the increasing incidence of vertebral fractures are complex.
- Early and scientific management of high-risk osteoporosis patients is necessary.

Osteoporotic fractures are a major public health problem worldwide, and dramatic population aging makes aging-related diseases more serious^1^. Vertebral fractures are one of the most serious consequences of osteoporosis and can cause heavy medical burdens^2^ and increased mortality risk for 5–10 years^3^. The all-cause mortality rate of vertebral fractures after 1 year is 3.1–10.04%^4,5^; however, after 3 years, the mortality rate rises to 46%^6^. Vertebral fractures were also recognized as sentinel events for injuries associated with higher morbidity and mortality. It has been reported that patients with hip fractures may be accidentally discovered to have old vertebral fractures^7,8^. In addition, patients with previous spine fractures were 7.3 times more likely to suffer another vertebral fracture than those without such a history^9^.

The epidemiology of vertebral fractures is less clear. There is no universally accepted definition and uniform diagnostic criteria, and it is difficult to distinguish old and new fractures based on the common X-ray radiographs alone^10^. In addition, due to their silent nature, vertebral fractures remain largely undermanaged and underappreciated^5,11^. There are few studies on the incidence and cost of vertebral fractures, especially in the past decade. Furthermore, existing studies are also mainly conducted in developed countries, with little data on developing countries.

The Urban Employee Basic Medical Insurance (UEBMI) and Urban Resident Basic Medical Insurance (URBMI) databases in China, as two major national population-based administrative databases, provide a good opportunity to evaluate the burden of vertebral fractures in China, the largest developing country in the world. In the UEBMI and URBMI databases, all reimbursements for vertebral fractures were recorded, and the medical expense for old vertebral and fresh vertebral fractures varied greatly; therefore, by taking the expense of patients into consideration, it is possible to distinguish old from acute fractures. Remarkably, high-energy injuries, such as accidental falls, car traffic accidents, and work injuries, were not covered by the UEBMI and URBMI programs, which can help preclude vertebral fractures not caused by osteoporosis.

This study aimed to assess the incidence of clinically recognized vertebral fractures and related medical costs among patients aged 50 years and over in China from 2013 to 2017 to further reveal the epidemiological characteristics of osteoporotic fractures in China.

## Material and methods

### Database and study population

This study used claims data from the Urban Employee Basic Medical Insurance (UEBMI) database and Urban Residence Basic Medical Insurance (URBMI) database in China. UEBMI and URBMI are the two main medical insurance programs for the Chinese urban population. The populations insured by UEBMI are retired and working employers/employees in cities. The populations insured by URBMI are unemployed people in cities, such as children, senior citizens, and students. The information included in the two databases is sociodemographic characteristics (i.e. gender, date of birth, residential address, ethnicity, etc.), diagnoses (disease names, disease codes, etc.), medical expenses, and so on. The reimbursement records of all insured people were kept in the two databases, even if the expense reimbursed was zero. The two databases are updated at the city level monthly. In 2016, the two databases covered over 95% of the Chinese population in urban areas^12,13^. However, a new system was designed to take on the increasing health big data, and medical administrations have yet to release new data from 2018. Therefore, only medical records of patients from 2013 to 2017 are available, up-to-date, and the most complete. In UEBMI and URBMI, patients may not be continuously insured. This was a dynamic cohort, with all people followed from getting insured to not paying the premium. The two databases have been used in some high-quality studies^14–16^, which confirmed the reliability of the two databases to some extent.

The study population came from 23 provinces covered by UEBMI and URBMI between 1st January 2013 and 31st December 2017. There are a total of 31 provinces in Mainland China, and the other 8 provinces in Mainland China were excluded (~20% of the urban Chinese population) due to missing or unqualified data such as diagnoses, information on coverage only by insurance programs, and reporting policy exemptions. This study was approved by the ethical review committee of the Peking University Health Science Center (IRB. No.: IRB00001052-18012), and the informed consent requirement was waived. This work has been reported in line with the STROCSS (Strengthening The Reporting of Cohort Studies in Surgery) criteria^17^, Supplemental Digital Content 1, http://links.lww.com/JS9/A416.

### Case identification

Patients with vertebral fractures were identified by the diagnostic test and International Classification of Diseases (ICD)-10 code of primary diagnoses in the databases. The ICD-10 codes had been widely used in UEBMI and URBMI during the study period, and many previous high-quality studies^16,18–21^ have also mentioned and used them. The original diagnostic text in the databases was unstructured; therefore, natural language processing was used to normalize it according to the vertebral fracture dictionary created by prestigious orthopedists. According to Chinese medical terms (i.e. ‘thoracic vertebral fracture’ and ‘lumbar vertebral fracture’) and ICD-10 codes (i.e. S22 and S32) for vertebral fractures, potential patients with vertebral fractures were identified first. To identify the actual target patients, each potential target patient was evaluated by two expert clinicians independently. Cohen’s kappa statistic of the two expert clinicians was 0.75, which suggests a good consistency (sTable 1, Supplemental Digital Content 2, http://links.lww.com/JS9/A417). Any disagreements between them are mediated by another senior orthopedist.

In this study, vertebral fractures were defined as clinically recognized vertebral fractures, and all patient data, including the evidence for diagnosis, demographic information, and medical costs, were completed for claims. In the clinical setting, clinically recognized vertebral fractures were referred to as those with symptoms of low back pain, and a complete radiological examination was interpreted as a spinal fracture.

To distinguish new vertebral fracture cases from old vertebral fracture cases, a distribution map of patient costs was compiled. Given experiences in clinical practice, most patients with the first occurrences of vertical fractures will be hospitalized, and even if not hospitalized, the outpatient service cost is higher than that in a return visit. According to the analyses and description of the claims data, 1000 renminbi (RMB) is an appropriate cutoff value. Therefore, new vertebral fractures were defined as outpatient visits for vertebral fractures with costs greater than 1000 RMB and hospitalizations for vertebral fractures.

### Statistical analysis

The incidence of clinically recognized vertebral fractures was calculated in two steps. In the first step, the annual incidence of each province was calculated, with the numerator as the number of vertebral fractures and the denominator as the person-years of each province in a certain year. Since a small portion of insured individuals in the UEBMI and URBMI databases had claim records with missing diagnostic information, the number of vertebral fractures needed to be estimated for these individuals. This number was estimated by assuming that the proportion of vertebral fractures among these individuals was equal to that among those with diagnostic information. The normality and homogeneity of the provincial incidence rates in 2013, 2014, 2015, 2016, and 2017 were tested by Skewness/Kurtosis tests and Cochran’s *Q* statistic (sTable 2, Supplemental Digital Content 2, http://links.lww.com/JS9/A417).

In the second step, a random-effects meta-analysis was used to pool the incidence of all 23 included provinces in calculating the national incidence; in this step, the Freeman–Tukey double arcsine transformation was used to stabilize the variance in province-specific incidence. Subgroup analysis was calculated by sex and age (50–59, 60–69, 70–79, and ≥80). The age-adjusted incidence in 2017 was calculated based on the standard population with the 2010 Chinese national census, Revised European Standard Population (RESP) 2013, 2010 U.S. population, and 2011 Australian population data. Two sensitivity analyses for incidence in 2017 were also performed: first by considering only the vertebral fractures among those who were not missing diagnostic information in their claim records to estimate the lower bound of the incidence and second by excluding the top 10% of provinces in terms of the missing rate of diagnostic information. Also, to avoid underestimating the incidence, another sensitivity analysis was conducted by canceling the threshold of 1000 RMB outpatient costs (sTable 3, Supplemental Digital Content 2, http://links.lww.com/JS9/A417).

The medical costs of clinically recognized vertebral fractures were also evaluated. Two types of medical/drug costs were evaluated: medical costs for vertebral fractures and drug costs for vertebral fractures. Additionally, the per capita costs of the above types were evaluated. The costs from 2013 to 2016 were also discounted by the consumer price index (CPI) to costs in 2017. All statistical analyses were performed by Stata version 15.0.

## Results

**Figure 1:**
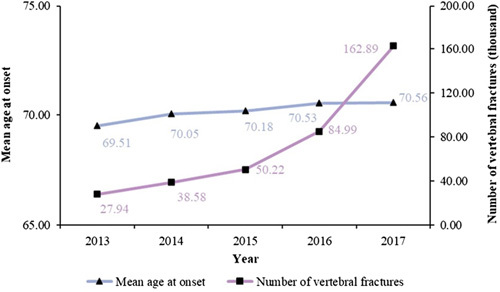
The mean age at onset and the number of clinically recognized vertebral fractures in urban China, 2013–2017.

This population-based cohort study was conducted by using UEBMI and URBMI data in China from 2013 to 2017, covering ~407.2 million person-years. Medical insurance data from 23 provinces in Mainland China were included. A total of 271 981 patients with clinically diagnosed vertebral fractures were included in this study, including 186 428 females and 85 553 males (Table 1). The mean age at first onset was 70.26 years (SD: 10.52) (Table 1). In detail, the number of vertebral fractures was 27.94 thousand in 2013 and 162.89 thousand in 2017, while the mean age of vertebral fracture patients was 69.51 in 2013 and 70.56 in 2017 (Fig. 1).

**Table 1:** Characteristics of patients with vertebral fractures in urban China, 2013–2017.

Characteristics	Male	Female	Total
Number	85 553	186 428	271 981
Mean age (years, SD)	69.03 (11.06)	70.83 (10.22)	70.26 (10.52)
Age group, *n* (%)			
50–59	19 719 (23.05)	28 887 (15.49)	48 606 (17.87)
60–69	24 795 (28.98)	54 697 (29.34)	79 492 (29.23)
70–79	23 085 (26.98)	60 494 (32.45)	83 579 (30.73)
≥80	17 954 (20.99)	42 350 (22.72)	60 304 (22.17)

### Incidence of vertebral fracture

**Figure 2:**
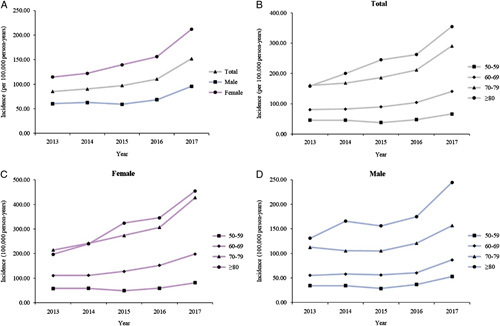
Incidence of clinically recognized vertebral fracture grouped by sex, age, and sex–age group (per 100 000 person-years). (A) Clinically recognized vertebral fracture incidence by sex and year. (B) Age-specific clinically recognized vertebral fracture incidence. (C) Age-specific clinically recognized vertebral fracture incidence for females. (D) Age-specific clinically recognized vertebral fracture incidence for males.

The incidence of clinically recognized vertebral fracture in 2017 was 152.13 per 100 000 person-years (95% CI: 93.86–224.38), with incidence among males and females of 95.70 (95% CI: 62.91–135.33) and 212.07 (95% CI: 125.91–320.50), respectively (Table 2 and Fig. 2). Regarding the age trend in incidence, the incidence peaked at 80+ years of age (Table 2 and Fig. 2).

**Table 2:** Incidence of clinically recognized vertebral fracture in urban China, 2013–2017.

	Incidence (per 100 000 person-years, 95% CI)				
Subgroup	2013	2014	2015	2016	2017
Total	85.21 (66.37–106.40)	90.37 (71.29–111.71)	97.35 (68.52–131.22)	110.49 (79.54–146.50)	152.13 (93.86–224.38)
Sex					
Male	60.42 (47.50–74.89)	62.78 (49.49–77.64)	59.27 (43.22–77.86)	68.32 (50.34–89.04)	95.70 (62.91–135.33)
Female	114.51 (87.14–145.60)	122.26 (95.34–152.50)	139.43 (95.80–191.20)	156.16 (110.28–209.99)	212.07 (125.91–320.50)
Age					
50–59	45.59 (36.47–55.73)	45.72 (36.70–55.74)	37.88 (27.53–49.89)	47.49 (34.55–62.48)	66.60 (42.26–96.45)
60–69	80.98 (63.31–100.82)	82.73 (65.42–102.07)	89.77 (64.75–118.85)	104.45 (74.94–138.84)	140.61 (90.48–201.73)
70–79	160.72 (118.81–208.93)	168.67 (129.73–212.70)	186.20 (130.32–252.00)	211.70 (152.90–280.02)	291.04 (176.60–433.81)
≥80	158.63 (110.97–214.76)	200.41 (145.53–264.03)	245.30 (154.41–357.06)	262.69 (177.14–364.99)	355.02 (199.07–555.56)

In terms of the temporal trend in incidence, the incidence remained relatively stable during the first 3-year period and gradually increased after 2015, although the 95% CIs overlapped. The incidence standardized by the 2010 Chinese national census data was slightly lower than the unstandardized incidence, but those incidence standardized by the RESP 2013, the 2010 U.S. population, and the 2011 Australian population were slightly higher than the unstandardized incidence (Table 3). The sensitivity analyses can be seen in Table 3.

**Table 3:** Standardization and sensitivity analysis of the clinically recognized vertebral fracture incidence in urban China, 2017.

	Incidence (per 100 000 person-years, 95% CI)		
	Male	Female	Total
Standardized incidence			
2010 Chinese national census data	91.88 (91.11–92.66)	198.14 (197.01–199.29)	144.17 (143.49–144.86)
RESP 2013 population census data	109.99 (109.15–110.84)	240.02 (238.77–241.28)	174.53 (173.78–175.29)
2010 US population census data	102.25 (101.44–103.06)	214.52 (213.34–215.71)	157.94 (157.22–158.66)
2011 Australian population census data	105.39 (104.57–106.22)	223.93 (222.72–225.14)	164.19 (163.46–164.92)
Sensitivity analysis			
Sensitivity analysis one^a^	68.93 (41.20–103.75)	159.01 (84.94–256.08)	112.62 (62.67–177.08)
Sensitivity analysis two^b^	101.78 (69.06–140.80)	226.16 (138.68–334.86)	161.99 (103.36–233.71)

RESP, Revised European Standard Population.

a Including only observed cases to assess the lower bounds of the rates.

b Excluding the top 10% of provinces with missing diagnostic information.

### Costs of vertebral fracture

**Figure 3:**
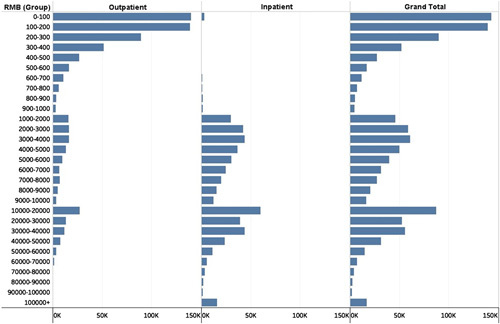
Cost distribution map for clinically recognized vertebral fracture in urban China, 2013–2017.

Real-world data about medical insurance payments and person counts for outpatient, inpatient, and total costs of vertebral fractures are calculated and displayed as bar graphs (Fig. 3).

**Figure 4:**
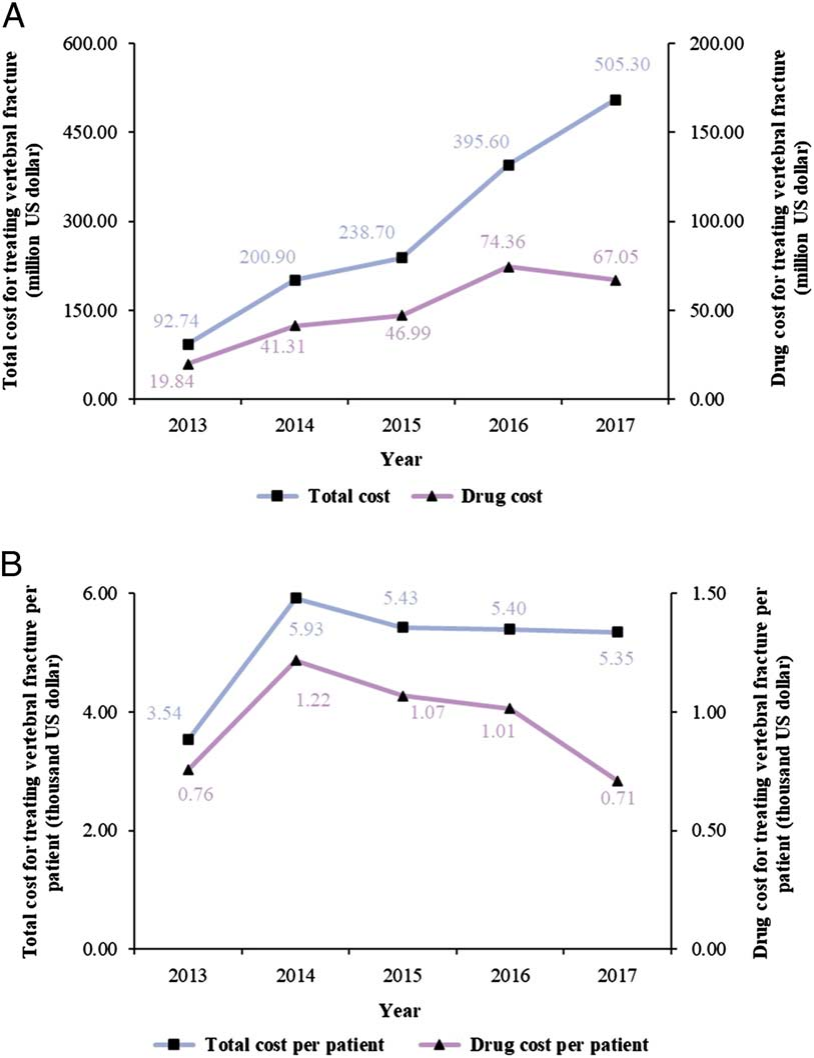
Annual costs for vertebral fracture from 2013 to 2017 (in U.S. dollars). (A) Total cost and drug cost for treating clinically recognized vertebral fracture; (B) total cost and drug cost for treating clinically recognized vertebral fracture per patient.

During the 5-year period from 2013 to 2017, the total medical cost for vertebral fractures increased from US$92.74 million to US$505.30 million (Fig. 4). The total drug cost increased from US$19.84 million to US$67.05 million. However, the total medical costs and drug costs for clinically recognized vertebral fractures per patient increased sharply in 2014 but remained flat thereafter.

## Discussion

This nationally representative population-based study found that the incidence of clinically recognized vertebral fractures in Mainland China increased ~1.79-fold, and the medical cost for vertebral fractures increased dramatically 5.45-fold from 2013 to 2017.

The incidence of clinical vertebral fractures is sharply increasing in urban China. China’s rapidly aging population creates more vertebral fractures. Although the prevalence of osteoporosis among people over the age of 50 was ~31% in 2007^22^ and 32% in 2018^23^, the absolute number of the elderly population has grown from 153 million in 2007 to 249 million in 2018, which increased ~1.6-fold during a short 10 years^24^. In addition to decreased skeleton quality, poor physical condition and the propensity to fall contribute to fracture risk with advancing age^25^.

An emphasis on health increases the health-seeking behaviors of patients, and an updated understanding of the severity of fragility fractures among doctors also places more emphasis on medical support for vertebral fractures, especially after China issued two important documents on the prevention and treatment of osteoporosis, ‘Key Points of Prevention and Treatment of Osteoporosis’ ^26^ and ‘Guidelines for Diagnosis and Treatment of Primary Osteoporosis’ ^27^ in 2011. Since then, the availability of medical imaging equipment and purposeful radiographic examinations has improved the diagnosis rate of vertebral fractures.

The incidence of vertebral fracture in urban China is still lower than that in developed countries (Table 4)^34,37^. One important reason for the high incidence in developed countries is that these countries have large proportions of elderly people in their population, and fall-caused vertebral fractures are more common in elderly people^38^. In addition, people in developed countries have higher education levels, health awareness, and more abundant medical resources than those in developing countries. Another reason is the differences between different races and new knowledge about bone microarchitecture and bone quality. Compared with Caucasians, Chinese individuals have lower area bone mineral density at both vertebral and nonvertebral sites. However, the Chinese skeleton has advantages in both cortical and trabecular compartments, which compensate for the lower bone strength and smaller bone size^39^.

**Table 4:** A literature review of the reported incidence of vertebral fractures around the world.

Year of publication	Study period	Country, city, region	Setting	Number of cases (men/women)	Results	Reference
1999	1999	Australia	Geelong Osteoporosis Study region	75/243	The incidence of vertebral fracture is 19/10 000 per year in women aged 35 years and over and 7/10 000 per year in men	^28^
2002	2002	Europe, 29 European centers	The European Vertebral Osteoporosis Study	14 011	The incidence of vertebral fracture is 10.7/1000 per year in European women aged 50 years and over and 5.7/1000 per year in men	^29^
2007	2002	Spain	Ministry of Health and Consumer Affairs’ Hospital Discharge Database	7100	The incidence of vertebral fracture is 11.9/10 000 per year in women aged 80 years and over and 8.9/10 000 per year in men	^30^
2008	2004	Japan, Sado City	A survey of patients at Sado General Hospital (inpatients and outpatients)	45/118	The incidence per 100 000 was 134.7 in men, 322.5 in women	^31^
2009	2000–2006	Swiss	Swiss Federal Office of Statistics	2622/6313	The incidence of spine fractures was 486 per 100 000 in the age group 50 years and older women, and 243 per 100 000 for men	^32^
2010	1997–2001	Sweden	Swedish Hospital Discharge Register	2801/1729	The incidence of thoracolumbar fractures was 97.2 per 100 000 in the age group 60 years and older in 1997 and 80.5 per 100 000 in 2001	^33^
2010	2004–2006	Japan, Sado City	A survey of patients at Sado General Hospital (inpatients and outpatients)	Total 163–188	The incidence per 100 000 was 232.8 in 2004, 246.9 in 2005, and 282.3 in 2006	^34^
2011	2005–2008	Korea	Health Insurance Review and Assessment Service database	Total 189 856–210 592	The incidence per 100 000 was 974 in 2005, 991 in 2006, 989 in 2006, and 956 in 2007	^35^
2012	2008–2012	Korea	Health Insurance Review and Assessment Service database	Total 14 808–79 903	The crude overall incidence of vertebral fractures was 984 per 100 000 person-years from 2005 to 2008	^36^
2016	2008–2012	Korea	National Health Insurance Service	Total 111 304–126 446	The incidence was 245.3/100 000 in men and 780.6/100 000 in women in 2008 and 312.5/100 000 in men and 953.4/100 000 in women in 2012	^37^

The incidence in females was nearly three times higher than that in males in this study, which is similar to previous studies regarding the incidence of vertebral fractures^10^. However, the result of The China Osteoporosis Prevalence Study (COPS) showed that the prevalence of vertebral fractures in males was close to that in females^23^. The possible explanation was that men are more likely to have vertebral fractures because of traffic accidents and labor work before the age of 50, while women are more likely to have vertebral fractures because of postmenopausal osteoporosis after the age of 50^38^. As a result, old vertebral fractures accounted for a higher proportion of vertebral fractures in men over 50 years of age, while new vertebral fractures accounted for a higher proportion of vertebral fractures in women over 50 years of age. Therefore, although the prevalence may be similar between men and women, the incidence should be higher in women than men among those aged 50 years and over.

The increasing cost of treating vertebral fractures in urban China increases the burden on the healthcare system. In detail, the total medical cost for vertebral fractures increased ~5.45-fold, while the cost per patient also increased 1.51-fold. The possible explanations are as follows. First, the total absolute number of vertebral fractures in those 50 years and older increased ~5.8-fold. Second, the expanded indications of kyphoplasty and vertebroplasty and recommendations about the use of anti-osteoporosis drugs can also explain this phenomenon. However, in view of the academic controversy over vertebroplasty in the treatment of osteoporotic vertebral fractures^40^ and the American Association of Clinical Endocrinologists/American College of Endocrinology (AACE/ACE) clinical practice guidelines for the diagnosis and treatment of postmenopausal osteoporosis^41^, administrators should calmly consider the trade-offs between the benefits of surgical treatment and medical costs while acknowledging that these costs may be sizeable. Therefore, screening^42^ and effective systemic community management of osteoporosis as a kind of chronic disease^43^ may be a low-cost solution to identify groups at high risk of osteoporosis and reduce the imminent risk of fracture^41^.

Of interest, although the cost for clinically recognized vertebral fracture per patient has remained flat since 2014, the downstream costs, including professional care for fracture-related complications, treatment of comorbidities, prevention of future fractures, and overall increased cost of healthcare, should also be premeditated^44^. The inclusion of anti-osteoporosis drugs in medical insurance and as preventive drugs is a strategy that can reduce the incidence of osteoporotic fractures^45^ and thus reduce the cost of bone fracture treatment. However, the best way to balance the benefits of pharmacological prevention and the cost of treatment remains controversial, and evidence from advanced health economics studies is needed.

Hip fractures and vertebral fractures are two representative types of osteoporotic fractures. Previous studies suggested that the incidence of hip fracture is stable^16^, but our research suggested that the incidence of vertebral fracture is increasing. Unlike hip fractures, which are characterized by the immediate disappearance of mobility and urgent need for hospitalization and surgery^16^, many vertebral fractures are asymptomatic and affect a larger number of people. In addition, the hip is prone to fracture due to lateral external force^46^, while the vertebral body is vulnerable to both longitudinal and lateral external forces. This also explains why falls account for the majority cause of hip fractures^47^ while only responsible for a quarter of vertebral fractures^10^. Most of the vertebral fractures are caused by daily activities^48,49^. Therefore, the effect of fall prevention guidelines in preventing vertebral fractures is limited, and vertebral fractures more directly reflect the severity of osteoporosis.

In fact, osteoporosis is a preventable and treatable disease with a slow progression. However, the treatment of mildly symptomatic osteoporotic vertebral fractures is always conservative. A Japanese study showed that only 18% of patients with clinical vertebral fractures received anti-osteoporosis drugs^50^. In China, only 0.3 and 1.4% of men and women with osteoporosis, respectively, receive anti-osteoporosis therapy^23^. China has a large population, and any inconspicuous health problem can have a very large social burden. Therefore, understanding the incidence and cost of vertebral fractures in China can raise awareness of osteoporosis and emphasize the need for prevention, which is currently of great significance in China.

Appropriate strategic adjustments and policy innovations in public health are necessary. With the development of the economy and the prolongation of life expectancy, the changing pattern of disease burden in developing countries, including China, a developing country with strong economic strength, may follow that in developed countries. Calculating the incidence of osteoporotic fractures in China and exploring its practical management mode can enlighten other developing countries in their health-related policies. Additionally, for all countries facing the challenge of aging, it is necessary to prepare in advance to cope with the age-related medical burden that has come or is about to come.

## Strengths and limitations

This study used a nationally representative sample from China to supply abundant and reliable information about the incidence and costs of vertebral fractures. However, this study also has several limitations. First, vertebral fractures were identified by diagnostic text and ICD-10 codes, but not by X-ray re-confirmation. Second, there existed a small portion of ICD-9 codes in UEBMI and URBMI during the study period. But the ICD-9 codes all appeared with diagnostic text instead of appearing alone; therefore, they had no impact on the results of this study. Third, a part of the trend toward increasing vertebral fractures with time might be due to greater recognition of fractures, but that is unlikely to account for the very large increase in the incidence of clinical vertebral fractures. Fourth, we only include the urban population but lack data on the rural population. In view of the backward medical knowledge and lack of medical resources in rural areas, future work should focus on large-scale screening and management of osteoporosis patients in rural areas.

## Conclusion

In this population-based study, the incidence and cost of clinically recognized vertebral fractures increased in China. As the population ages, China will face an ever-increasing burden from vertebral fractures, so more attention should be given to the management of osteoporosis patients and the prevention of osteoporotic fractures.

## Ethical approval

This study was approved by the ethical review committee of the Peking University Health Science Center (IRB. No.: IRB00001052-18012), and the informed consent requirement was waived.

## Sources of funding

This work was supported by the National Key Research and Development Program (2020YFC2009004, 2021YFC2501700), the National Natural Science Foundation of China (81874010, 82272554), and the PKU-Baidu Fund (2020BD014).

## Author contribution

X.-Q.Z. L.X. J.-x.W., S-.Y.Z. and C.-L.S.: conceptualization; All authors: data curation; X.-Q.Z. L.X. J.-x.W. S-.Y.Z. and C.-L.S.: formal analysis; C.-L.S.: funding acquisition; X.-Q.Z., L.X., J.-x.W., S-.Y.Z., and C.-L.S.: investigation; X.-Q.Z., L.X., J.-x.W., S-.Y.Z., and C.-L.S.: methodology; J.-x.W., S-.Y.Z., and C.-L.S.: project administration; J.-x.W., S-.Y.Z., and C.-L.S.: resources; X.-Q.Z., L.X., and J.-x.W.: software; S.R.C., W.-B.X., J.-x.W., S-.Y.Z. and C.-L.S.: Supervision; X.-Q.Z., L.X., J.-x.W., S-.Y.Z., and C.-L.S.: validation; All authors: visualization; X.-Q.Z., L.X., J.-x.W. S-.Y.Z. and C.-L.S.: writing – original draft; All authors: writing – review and editing.

## Conflicts of interest disclosure

The authors declare that they have no conflicts of interest.

## Research registration unique identifying number (UIN)

1. Name of the registry: Chinese Clinical Trial Registry (ChiCTR).
2. Unique identifying number or registration ID: ChiCTR1800018217.
3. Hyperlink to the registration (must be publicly accessible): http://www.chictr.org.cn/showproj.aspx?proj=30768

## Guarantor

Xuan-Qi Zheng and Chun-Li Song.

## Data availability statement

The data that support the findings of this study are available from the National Healthcare Security Administration of China, but restrictions apply to the availability of these data, which were used under license for the current study, and so are not publicly available. Data are, however, available from the authors on reasonable request and with permission of the National Healthcare Security Administration of China.

## Provenance and peer review

Not commissioned, externally peer-reviewed.

## Supplementary Material
