# Supplementary material for: Incidence and cost of vertebral fracture in urban China: a 5-year population-based cohort study
Source: Int J Surg. 2023 May 3;109(7):1910–8. doi: 10.1097/JS9.0000000000000411 (PMC10389405; doi:10.1097/JS9.0000000000000411)
Supplement: Supplementary file 2 [file js9-109-1910-s002.docx]

**Supplementary Tables**

**sTable 1. The judgement results of the two experts**

| **Expert** |  | **B (N)** | | **Total (N)** |
| --- | --- | --- | --- | --- |
|  |  | **Not VFx** | **VFx** |  |
| **A (N)** | **Not VFx** | 10,013 | 3,603 | 13,616 |
|  | **VFx** | 2,218 | 77,324 | 79,542 |
| **Total (N)** | | 12,231 | 80,927 | 93,158 |

**Note: VFx= Vertebral Fracture**

**sTable 2.** **The normality and homogeneity of the provincial incidence rates in 2013, 2014, 2015, 2016, and 2017**

| **Year** | **Normality ^a^** | | **Homogeneity ^b^** | |
| --- | --- | --- | --- | --- |
|  | **Adjusted** $\boldsymbol{\chi}^{\boldsymbol{2}}$ | ***P* value** | **Q statistic** | ***P* value** |
| 2013 | 6.61 | 0.0367 | 16622.04 | <0.001 |
| 2014 | 4.34 | 0.1139 | 28199.90 | <0.001 |
| 2015 | 25.75 | <0.0001 | 40464.75 | <0.001 |
| 2016 | 4.74 | 0.0935 | 54293.72 | <0.001 |
| 2017 | 11.22 | 0.0037 | 129633.34 | <0.001 |

^a^ Skewness/Kurtosis tests; ^b^ Cochran’s *Q* statistic.

**sTable 3. The incidence of clinically recognized vertebral fracture in urban China, 2013–2017**

|  | **Incidence (per 100,000 person-years, 95% CI)** | | |
| --- | --- | --- | --- |
| **Year** | **Without 1,000 RMB threshold** | **With the 1,000 RMB threshold** | |
| 2013 | 112.73 (83.40 to 146.46) | | 85.21 (66.37 to 106.40) |
| 2014 | 117.85 (82.08 to 160.05) | | 90.37 (71.29 to 111.71) |
| 2015 | 117.39 (78.47 to 164.11) | | 97.35 (68.52 to 131.22) |
| 2016 | 132.60 (92.58 to 179.77) | | 110.49 (79.54 to 146.50) |
| 2017 | 165.79 (117.04 to 222.98) | | 152.13 (93.86 to 224.38) |
